# PSMA-Targeted Supramolecular Nanoparticles Prepared From Cucurbit[8]uril-Based Ternary Host–Guest Recognition for Prostate Cancer Therapy

**DOI:** 10.3389/fchem.2022.847523

**Published:** 2022-02-10

**Authors:** Xueyan Zhang, Shaolong Qi, Dahai Liu, Jianshi Du, Jingji Jin

**Affiliations:** ^1^ School of Life Sciences, Jilin University, Changchun, China; ^2^ Lymph and Vascular Surgery Department, China-Japan Union Hospital of Jilin University, Changchun, China

**Keywords:** prostate cancer, drug delivery, nanomedicine, host–guest complex, supramolecular chemistry

## Abstract

Nanomedicines play an important role in cancer therapy; however, some drawbacks including unsatisfactory efficacy and side effects arising from indiscriminate drug release retard their clinical applications. Although functionalization of nanomedicines through covalent interactions can improve the pharmacokinetics and efficacy of the loaded drugs, complicated and tedious synthesis greatly limits the exploration of multifunctional nanoparticles. Herein, we utilize a supramolecular strategy to design a nanomedicine for targeted drug delivery through cucurbit[8]uril-based host–guest ternary complexation and successfully prepare prostate-specific membrane antigen (PSMA)-targeted supramolecular nanoparticles encapsulating doxorubicin (DOX). *In vitro* studies exhibit targeted modification via noncovalent enhance anticancer efficiency of DOX due to the increased cell uptake on account of receptor-mediated endocytosis. This design provides a new strategy for the development of sophisticated drug delivery systems and holds perspective potentials in precise cancer treatments.

## Introduction

In order to overcome the inherent shortcomings of traditional chemotherapy using anticancer drugs with small molecular weight, nanotechnology has attracted a lot of attention and been extensively applied in drugs delivery to promote anticancer efficacy (Peer et al., 2007; Wicki et al., 2015; Shi et al., 2017; Wu D. et al., 2021). Nanomedicines hold tremendous advantages, such as enhanced water solubility, low immunogenicity and cytotoxicity, prolonged circulation time in plasma, and controllable drug release, which improve efficacy and reduce side effects (Davis et al., 2008; Kim et al., 2010; Petros and DeSimone, 2010; Colson and Grinstaff, 2012; Torchilin, 2014; Shreffler et al., 2019; Parodi et al., 2021). Several therapeutic nanoplatforms have been advanced in clinical trials, and some of them have been licensed for clinical cancer therapy, including liposomes, albumin nanoparticles (NPs), and polymer NPs (Green et al., 2006; Kratz, 2008; Kamaly et al., 2012; Allen and Cullis, 2013). Moreover, passive targeting based on the enhanced permeability and retention (EPR) effect increases the drug concentrations in the cancer environment. However, it is difficult to achieve the expectative therapeutic effect only by passive targeting (Matsumura and Maeda, 1986; Maeda et al., 2000; Fang et al., 2011; Park et al., 2019). To enhance the targeting performance of NPs, targeted nanomedicines were designed to typically integrate cancer-specific recognition motifs including antibodies, peptides, and small molecular ligands into the NPs via covalent bonds by chemical coupling, usually accompanied by complicated synthesis, high cost, and potential cytotoxicity (Bander et al., 2005; Yu et al., 2010; Manivasagan et al., 2019; Rosellini et al., 2021). How to modify NPs simply and reliably by optimizing the preparation method in order to improve the property of drugs is a research topic of great interest.

Supramolecular chemistry is applied to binding two or more species into complexes with specific structure and properties through intermolecular interactions (Lehn, 1988; Zhou et al., 2021). Benefiting from the advantages of noncovalent interactions including van der Waals, metal coordination, hydrogen bond, hydrophobic interactions, and host–guest interactions, supramolecular architectures are capable of reversibly combining several functional blocks, such as targeted ligands, responsive moieties, and imaging groups separately into a single platform, which exhibit promising potential for drug delivery (Ling et al., 2008; Adler-Abramovich and Gazit, 2014; Kopp et al., 2017; Price and Gibson, 2018; Zhou et al., 2019). Furthermore, nanomedicines designed by host–guest chemistry holds stimuli-responsive capability, in which the non-covalent linkages are sensitive to specific tumor microenvironment, facilitating drug accumulation and release at the sites of action (Appel et al., 2012; Busseron et al., 2013; Webber et al., 2016; Han et al., 2018; Yu and Chen, 2019). The host–guest molecular recognition based on cucurbit[8]uril (CB [8]) is very promising because CB [8] can accommodate parallel π–π stacking geometry of electron-sufficient donors and electron-deficient acceptors in its large cavity (479 Å^3^), which means CB [8] is able to form a 1:1:1 ternary host–guest complex serving as a noncovalent linker (Barrow et al., 2015; Pazos et al., 2019; Sun et al., 2021; Wu H. et al., 2021). In the development of supramolecular nanomedicines, CB [8] holds the ability to integrate targeted molecules into the system through non-covalent interactions (Wu et al., 2017). Furthermore, the stability and biosafety of molecular recognition based on CB [8] have been proven *in vivo* (Wu H. et al., 2021). All those excellent nature makes CB [8] stand out from other supramolecular materials in the field of supramolecular nanomedicine.

**SCHEME 1:**
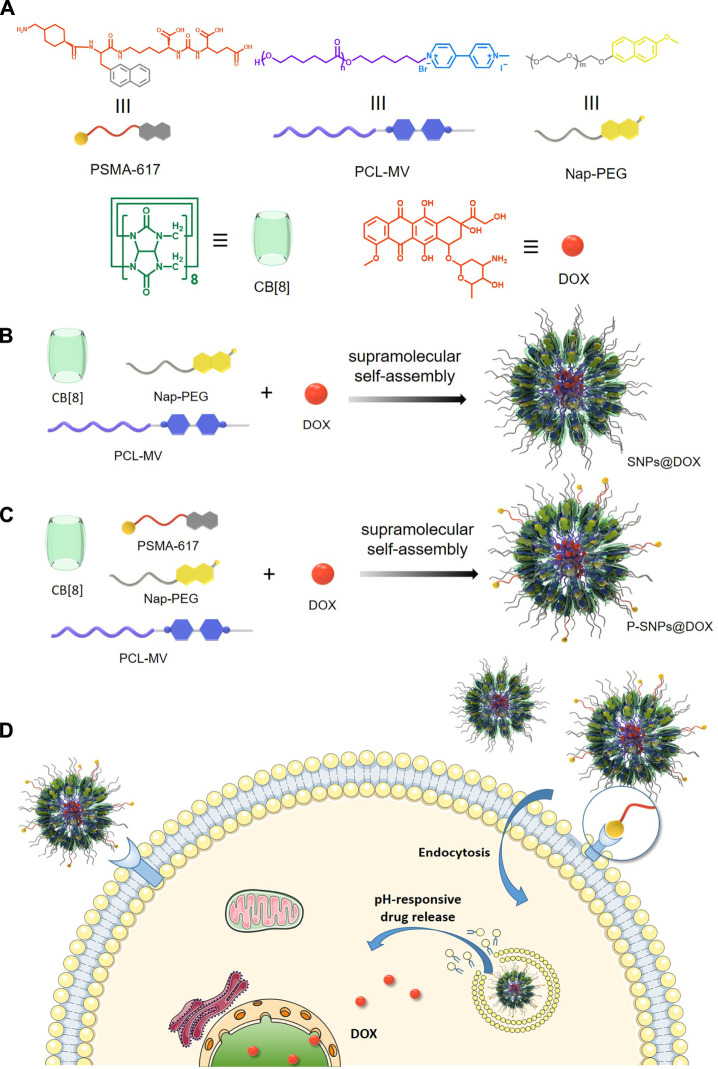
**(A)** The chemical structures of PSMA-617, Nap-PEG, PCL-MV, CB [8], and DOX. **(B)** The preparation of supramolecular nanomedicine SNPs@DOX. **(C)** The preparation of supramolecular nanomedicine P-SNPs@DOX. **(D)** Schematic of drug delivery and cellular internalization of SNPs@DOX and P-SNPs@DOX.

Herein, we integrate a targeted ligand into supramolecular NPs by using CB [8] host–guest molecular recognition. We design and synthesize two polymers as methylviologen (MV) linked with poly (ε-caprolactone) (PCL-MV) and naphthalene linked with polyethylene glycol (Nap-PEG). PCL-MV and Nap-PEG form a supramolecular amphiphile through ternary host–guest complexation between MV, Nap, and CB [8] (Scheme 1). Intriguingly, prostate-specific membrane antigen (PSMA)-617 as a prostate cancer targeting ligand contains a naphthalene group, which allows it to insert into the cavity of CB [8] together with MV, thus endowing the supramolecular NP with targeting ability (Wright et al., 1995; Kratochwil et al., 2016; Feuerecker et al., 2021; Plichta et al., 2021). According to the molecular recognition mentioned above, we have successfully prepared doxorubicin (DOX)-loaded NPs (SNPs@DOX) and PSMA-targeted DOX-loaded NPs (P-SNPs@DOX). Then we prove both P-SNPs@DOX and SNPs@DOX are internalized by 22RV1 and PC3 cells, while P-SNPs@DOX has a higher cellular uptake by 22RV1 as PSMA-positive cells. Moreover, *in vitro* experiments exhibit that P-SNPs@DOX shows higher cytotoxicity against 22RV1 cells. These studies suggest that the introduction of PSMA enables nanomedicines to be specifically internalized by cancer cells overexpressing PSMA, thereby increasing the anticancer efficacy. In addition, we also verify that the supramolecular NPs we designed can encapsulate other hydrophobic drugs, such as paclitaxel (PTX), proving the system is universally applicable.

## Materials and Methods

### Materials

ε-Caprolactone, 6-bromo-1-hexanol, *p*-toluenesulfonyl chloride, and methoxyl polyethylene glycol (mPEG-OH) were purchased from Sigma-Aldrich. Reagents are used without purification. Millipore ultrapure water was obtained on a Milli-Q purification system. MV-PCL and Nap-PEG synthesis method referenced published literature (Wu H. et al., 2021), with the same characterization parameters.

## Methods

### Preparation of SNPs@DOX and P-SNPs@DOX

DOX (6.00 mg), Nap-PEG (10.0 mg), and PCL-MV (15.0 mg) were dissolved in dimethyl sulfoxide (DMSO) (4 ml). About 20 ml of aqueous solution containing CB [8] (1.00 mg/ml) was added into the DMSO solution dropwise. After stirring in dark for 2 h, the obtained product was sealed in dialysis bags [molecular weight cutoff (MWCO) = 3.5 kDa] and dialyzed against deionized water for 12 h in order to remove free DOX and redundant CB [8]. The actual drug loading content was determined to be 10.5% by using ultraviolet spectroscopy.

For the preparation of PSMA-targeted nanomedicine, PSMA-617 (0.200 mg), DOX (6.00 mg), Nap-PEG (10.0 mg), and PCL-MV (15.0 mg) was dissolved in DMSO (4 ml). The subsequent preparation was conducted on the procedure mentioned above.

### Characterization of SNPs

The morphological characteristics and size were measured by transmission electron microscopy (TEM) (HITACHI, HT7700, Japan). Before measurements, the SNPs@DOX and P-SNPs@DOX were made evenly distributed on a carbon membrane-coated copper grid and completely dry. As to dynamic light scattering (DLS) by a DLS analyzer (Zetasizer Nano ZS90, Malvern, UK), SNPs@DOX and P-SNPs@DOX were appropriately diluted with water and then equilibrated at room temperature for 10 min and measured at different hour intervals in triplicate. UV absorption spectroscopy (Kratos Ltd., Manchester, UK) was carried out with a microplate reader (BioTek EPOCH 2, USA) using an optical source from 300 to 700 nm to determine the absorption light of aqueous solution including CB [8], MV, and Nap. Isothermal titration calorimetry (ITC) experiments were carried out with a VP-ITC microcalorimeter (Malvern, USA) at 298.1 K.

### Drug Release Studies

To monitor the release behavior of DOX from SNPs@DOX at different pH value, SNPs@DOX in 1 ml phosphate buffer saline (PBS) was sealed in dialysis bags (MWCO = 2 kDa), which were then put in 500 ml of the PBS solution at pH 7.4, 6.0, and 5.0 in triplicate, separately. The dialysis systems were stirred at 100 rpm at 37 C on magnetic stirrers. At predetermined time intervals, 100 μl of SNPs@DOX was taken out from the dialysis bag. The content of DOX was measured at 490 nm using microplate reader (BioTek EPOCH 2, USA) at room temperature. And then the medium was put back to the original solution in dialysis bag after being measured. According to the pre-measured standard curve, the amount of DOX released from nanomedicines was calculated with optical density (OD) value. All release experiments were carried out in triplicate, and all data were averages of nine determinations used for drawing figures.

### Prostate Cancer Cells Culture Conditions

22RV1 and PC3 cells were cultured in RPMI-1640 supplemented with 10% fetal bovine serum (FBS) and 1% penicillin-streptomycin (50 units/ml, P/S) at 37 C in 5% CO_2_ using humidifying cell incubator. The cells were digested and separated by trypsin (0.5% w/v in PBS, incubated for 2 min). The cell suspension was collected and centrifuged at 1,000 rpm for 5 min after being neutralized by perfect medium. The supernatant was discarded, and then the cells were resuspended in serum-supplemented RPMI-1640. The cells were seeded into cell culture dishes or suitable containers at an appropriate concentration to prepare for subsequent experiments.

### Cytotoxicity Assessment

The cytotoxic potential of SNPs, DOX, SNPs@DOX, and P-SNPs@DOX against PC3 cells and 22RV1 cells were determined by 3-(4′,5′-dimethylthiazol-2′-yl)-2,5-diphenyl tetrazolium bromide (MTT) assay in 96-well cell culture plates. Cells at a density of 1.5 × 10^5^ cells/ml (PC3) and 3 × 10^5^ cells/ml (22RV1) were seeded 100 μl to each well in a 96-well plate. After being cultured overnight, cells were then incubated by suspensions of SNPs, DOX, SNPs@DOX, and P-SNPs@DOX at equivalent DOX concentrations for 24 h or 48 h, in quintuplicate. And then the supernatant was removed and replaced by 100 μl/well of MTT solution (0.5 mg/ml) at indicated time intervals, and cells were incubated at 37 C for another 4 h. About 100 μl DMSO was added into each well with low-speed oscillation after discarding the original solution containing MTT. The absorbance of the formazan product was measured at 570 nm using a microplate reader. Untreated cells in medium were used as a control. According to the OD value obtained, cell viability = OD _treated_/OD _control_. The IC_50_ value was fitted by the *in vitro* cytotoxicity data

### Cellular Internalization Studies

Detection of the cell internalization implement was done through a LSM980 Airyscan2 confocal laser scanning microscope (CLSM) (Zeiss, Germany). 22RV1 (2 × 10^5^ cells/well) and PC3 (1 × 10^5^ cells/well) were seeded into glass-bottom cell culture dishes (∅20 mm, NEST). After 24 h, cells were treated with SNPs@DOX and P-SNPs@DOX (2.0 μM of DOX content) in 1640 medium at 37 C for 4 and 9 h, respectively. The cells were washed with PBS three times and immobilized with fresh 4.0% formaldehyde for 20 min at room temperature. After being rinsed with PBS three times, the cells were sealed with blocking solution containing DAPI dye (1 μg/ml). Finally, the dishes were observed with a CLSM (×40 oil objective, 401/577 nm excitation).

### Statistical Analyses

Statistical analyses were performed using GraphPad Prism 8.0. A *p*-value less than 0.05 was considered statistically significant. Data were presented as the mean ± standard deviation.

## Results and Discussion

### Characterizations of Host–Guest Complexation

**FIGURE 1:**
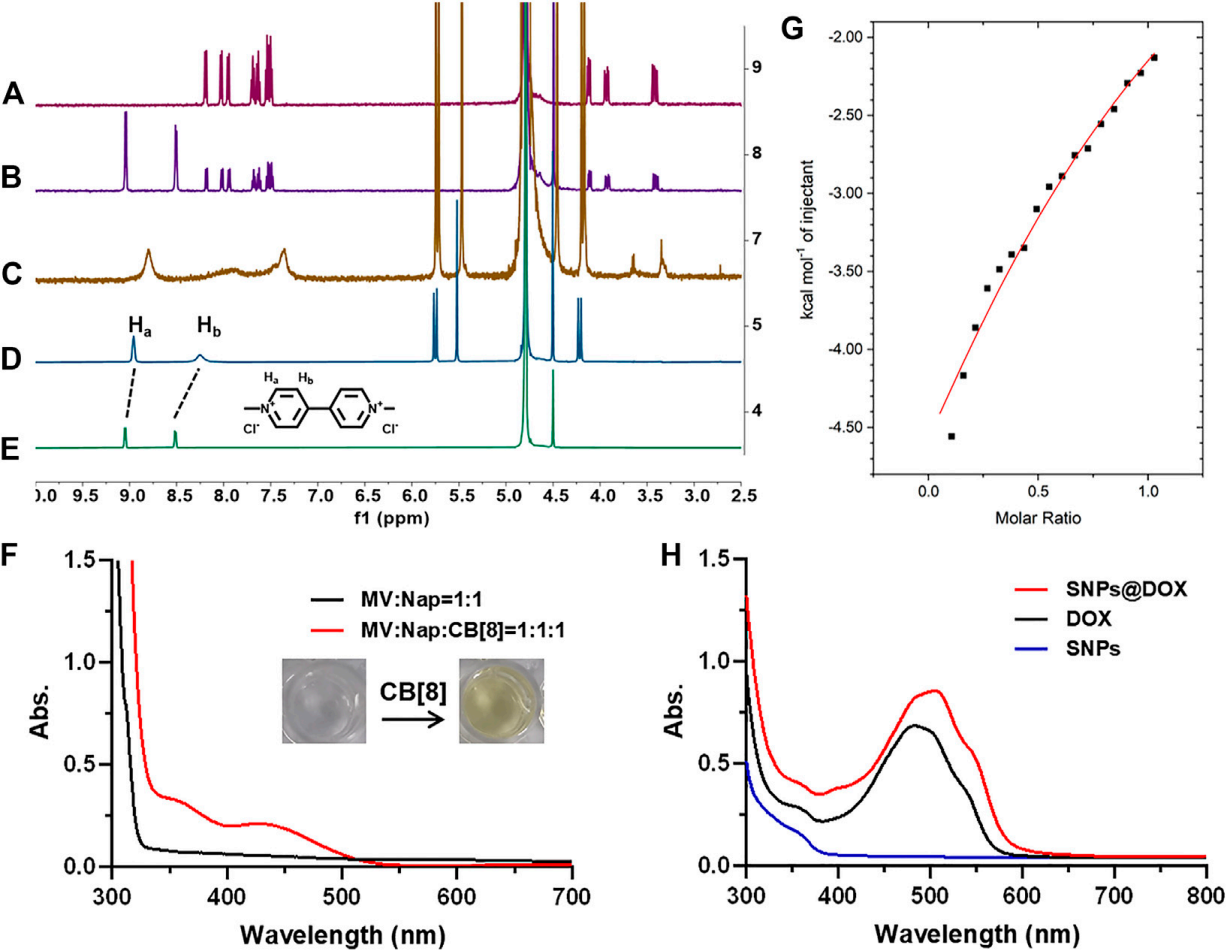
^1^H NMR spectra of **(A)** Nap, **(B)** Nap + MV, **(C)** Nap + MV + CB [8], **(D)** MV + CB [8], and **(E)** MV in D_2_O. **(F)** UV–vis spectra of the solution containing Nap (2.00 mM) and MV (2.00 mM) with/without CB [8] (2.00 mM). **(G)** ITC data and fitting curve of Nap (1.00 mM) titrated into the solution of CB [8] (0.100 mM) and MV (0.100 mM) in aqueous solution at 298.1 K. **(H)** UV–vis spectra of the solution containing DOX (1.00 mM), SNPs, and SNPs@DOX, respectively.

In order to characterize the ternary host–guest complexation, 2-amino-3-(naphthalen-1f-yl)propanoic acid (Nap) and MV were used as model guests. ^1^H NMR spectroscopy was utilized to characterize the host–guest interactions between CB [8], MV, and Nap (Figures 1A–E). As shown in the ^1^H NMR spectra, no obvious changes in chemical shift were observed for the solution containing MV and Nap, indicating the free state of these two joint molecules in the solution of D_2_O without CB [8] (Figure 1B). By virtue of the hydrophobic cavity and electron-sufficient carbonyl portals of CB [8], the dicationic guest MV could deeply thread into the host. As a result, MV showed upfield shift changes of its aromatic protons (H_a_ and H_b_) along with the addition of CB [8] (Figure 1D,E). As for a 1:1:1 mixture of MV, Nap and CB [8], significant changes in both shape and chemical shift of the peaks were monitored (Figure 1C). The splitting details of aromatic protons on Nap disappeared due to the broadened effect, and the proton signals of CB [8] shifted upfield, demonstrating the formation of a stable ternary host–guest complex. In order to further confirm the interactions in the ternary complex, 2D nuclear Overhauser effect spectroscopy (NOESY) NMR was performed in D_2_O (Supplementary Figure S4). NOE correlations were observed between the signals of aromatic protons on Nap, methyl protons on MV, and protons on CB [8], suggesting the insertion of MV and Nap into the cavity of CB [8], which was inconsistent with the results in ^1^H NMR spectra.

The UV–vis absorption spectrum further proved the generation of charge transfer (CT) interactions in the cavity of CB [8] (Figure 1F). Different from the mixture of Nap and MV in aqueous solution, upon adding the addition of CB [8] to the solution containing Nap and MV, a broad absorption band from 400 to 500 nm was observed, which was in accord with the representative CT band (Wu H. et al., 2021) Because the concentration of MV, Nap, and CB [8] used in preparation of SNPs@DOX was low, the absorption related to the CT band was not so apparent (Figure 1H). Moreover, the color of the solution immediately turned to light yellow upon addition of CB [8] into the solution containing Nap and MV (molar ratio = 1:1:1), which directly proved the formation of CT complex (Figure 1F). ITC was used to determine the association constants (K_α_) of the ternary complex which provided the thermodynamic behavior of the complex and binding affinity. The K_1_ value between CB [8] and MV was determined to be (1.53 ± 0.05) × 10^6^ M^−1^ using ITC measurement (Wu H. et al., 2021). By titrating the Nap solution into the solution of CB [8] and MV, the K_2_ value was calculated to be (7.87 ± 1.82) × 10^3^ M^−1^ (Figure 1G), verifying the ternary complexation. These studies showed that the interaction of the 1:1:1 ternary complex between CB [8], Nap, and MV was extremely stable. The strong non-covalent interactions provided the prerequisites for assembling NPs by using CB [8], derivative of MV (MV-PCL), and Nap (Nap-PEG) (Barrow et al., 2015).

### Fabrication of Supramolecular Nanoparticles and *In Vitro* Studies

**FIGURE 2:**
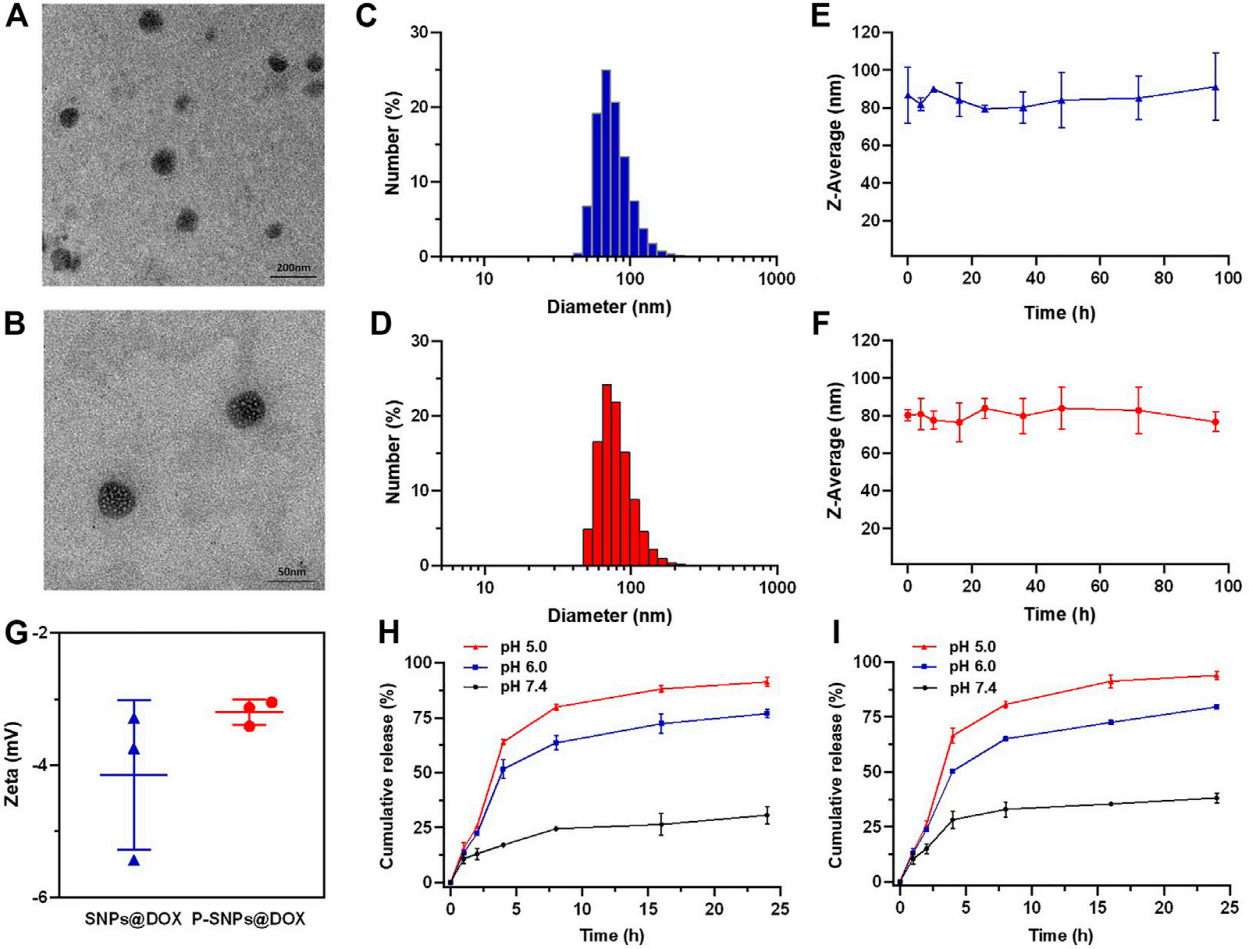
TEM images of **(A)** SNPs@DOX and **(B)** P-SNPs@DOX. DLS results of **(C)** SNPs@DOX and **(D)** P-SNPs@DOX in aqueous solution. The average diameters of **(E)** SNPs@DOX and **(F)** P-SNPs@DOX. **(G)** Zeta potential of SNPs@DOX and P-SNPs@DOX in aqueous solution. Release profiles of DOX from **(H)** SNPs@DOX and **(I)** P-SNPs@DOX at different pH values.

On the basis of molecular recognition, we used CB [8], Nap-PEG, and PCL-MV to construct a supramolecular copolymer, in which the hydrophobic and hydrophilic parts were connected by non-covalent bond in the cavity of CB [8]. Due to the amphiphilic property, NPs with a hydrophobic core were self-assembled from the supramolecular copolymers, which could load hydrophobic drugs, such as DOX. Actually, we successfully obtained the DOX-loaded supramolecular NPs (SNPs@DOX) with a drug loading content of 10.5% (Figure 1H). And we also managed to encapsulate PTX in this system (Supplementary Figures S14 and S15). In addition, we selected PSMA-617 containing naphthalene as the targeted ligand and successfully constructed PSMA-targeted NPs encapsulating DOX (P-SNPs@DOX) with a drug loading content of 11.7%. The drug loading of P-SNPs@DOX was comparative to that of SNPs@DOX, suggesting that package efficiency of DOX was almost unaffected by introducing PSMA-617. Figures 2A, B indicate that SNPs@DOX and P-SNPs@DOX are regularly round, with smooth surfaces. Their size was 60–110 nm (SNPs@DOX) and 50–70 nm (P-SNPs@DOX), respectively, as observed by TEM. DLS was used to reveal the average diameter of the NPs. The PDI values of SNPs@DOX and P-SNPs@DOX were less than 0.25. Figures 2C, D show that the average diameter was 86.8 nm (SNPs@DOX) and 80.3 nm (P-SNPs@DOX), slightly bigger than the size obtained from TEM studies because of the swelling effect. In addition, the average diameter of the NPs remains basically constant within 96 h incubation in PBS (Figures 2E, F). Furthermore, the zeta potentials of SNPs@DOX and P-SNPs@DOX were determined to be −4.15 and −3.2 mV, respectively (Figure 2G). These results demonstrated that SNPs@DOX remained stable in aqueous solution, and the introduction of PSMA-617 had no obvious effect on the stability of NPs. The simple synthesis and stability system constructed from noncovalent host–guest interactions would be conducive to the biomedical application of P-SNPs@DOX.

### *In Vitro* Drug Release

The release behaviors of DOX from SNPs@DOX and P-SNPs@DOX were observed at pH 7.4, 6.0, and 5.0, respectively, which simulated the pH gradient from normal to cancer tissue and blood to lysosome (Figures 2H, I). We verified that SNPs@DOX remained stable in PBS at pH 7.4, only releasing a small amount within 24 h. The release amount observably accelerated at low pH value, 76.9% at pH 6.0 and 91.4% at pH 5.0, respectively (Figure 2H). This phenomenon was due to the amine groups of DOX which could be protonated in an acidic environment. Compared with the release amount of DOX from SNPs@DOX, that of P-SNPs@DOX slightly increased in exactly the same conditions, 38.1% at pH 7.4, 79.6% at pH 6.0, and 93.9% at pH 5.0, which was conducive to DOX release to improve the efficacy of P-SNPs@DOX (Figure 2I). Depending on the deterministic nature of DOX, excessive release of DOX from SNPs@DOX and P-SNPs@DOX in acidic tumor microenvironments and intracellular lysosomes could improve drug concentration around the lesion.

### Cellular Uptake

**FIGURE 3:**
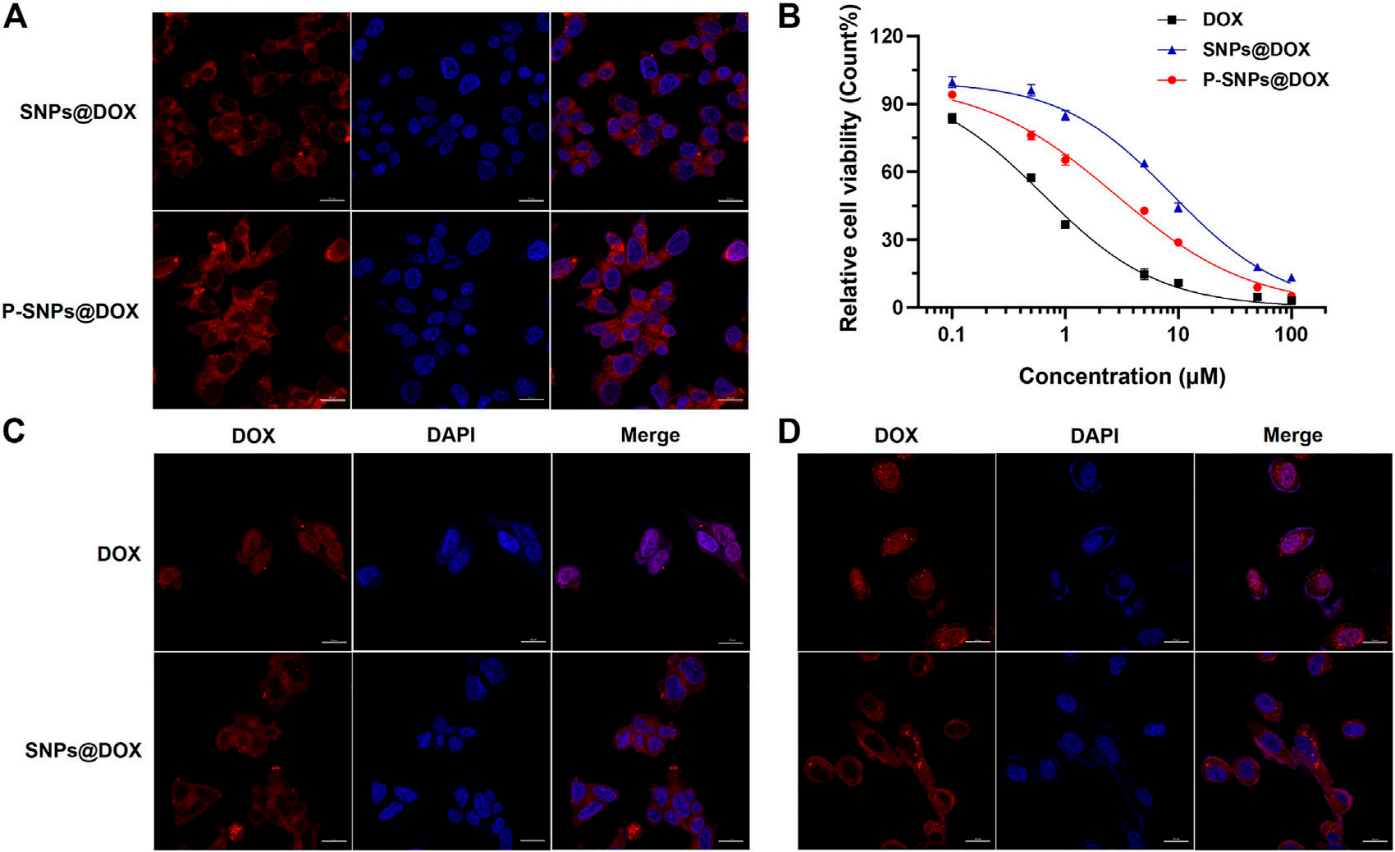
**(A)** CLSM images of 22RV1 cells cultured with SNPs@DOX and P-SNPs@DOX for 9 h, respectively. **(B)** Cytotoxicity of DOX, SNPs@DOX, and P-SNPs@DOX against 22RV1 cells for 48 h. CLSM images of **(C)** 22RV1 cells and **(D)** PC3 cells cultured with DOX and SNPs@DOX for 9 h, respectively.

It had been documented that the internalization and sustained retention of drug-loaded NPs in cancer cells could improve the therapeutic effect of drugs (Loureiro et al., 2016). In order to prove the advantages of DOX-loaded nanomedicine over DOX, we conducted *in vitro* studies to verify the internalization of prostate cells. CLSM was used to reveal the cellular uptake of SNPs@DOX. Supplementary Figures S7 and S8 exhibited red signals in the cytoplasm of 22RV1 and PC3 cells after 4 h incubation. By prolonging the culture time to 9 h, some fluorescent signals arise in the nucleus (Figures 3C, D). Free DOX was mainly located in cell nuclei after uptake, while SNPs@DOX and P-SNPs@DOX were located in the cytoplasm by endocytosis, indicating these nanomedicines could be effectively internalized into the cytoplasm of prostate cells. In contrast with SNPs@DOX, P-SNPs@DOX was more significantly ingested into 22RV1 cells, which was of great significance to targeted prostate cancer therapy (Figure 3A).

### *In Vitro* Cytotoxicity

In order to evaluate the superiority of P-SNPs@DOX over SNPs@DOX on killing prostate cancer cell *in vitro*, we used MTT assay to assess the cytotoxicity of the nanomedicines. Even at relatively high concentrations, supramolecular NPs composed of CB [8], Nap-PEG, and PCL-MV (SNPs) had little effect on cell viability without loading DOX, which indicated the nanomaterials we chose had excellent biocompatibility (Supplementary Figures S5 and S6). Figure 3B shows the relationship between the survival rate of the cells and the drug concentration after 48 h incubation. The cytotoxicity of DOX and drug-loaded NPs against prostate cancer cells was time- and concentration-dependent. Compared with the difference of endocytosis and slow drug release (Figure 3B). And a similar phenomenon also existed in PTX-loaded NPs (Supplementary Figures S15 and S16). Based on these results, we calculated the half-maximal inhibitory concentration (IC_50_) values of the nanoformulations. For 22RV1 cells, after 24 and 48 h incubation, the IC_50_ values of SNPs@DOX for 22RV1 cells were determined to be 26.5 ± 2.51 and 8.80 ± 0.70 μM, respectively (Figure 3B and Supplementary Figure S9). According to IC_50_ values of P-SNPs (10.4 ± 1.17 μM for 24 h and 2.77 ± 0.22 μM for 48 h), the cytotoxicity of P-SNPs@DOX exceeded that of SNPs@DOX (Supplementary Figure S13). This result indicated that the introduction of PSMA-617 increased the cytotoxicity of supramolecular nanomedicine to 22RV1 cells excluding PC3 cells, due to the recognition of PSMA-617 with PSMA on the surface of 22RV1 cells that effectively improved the efficiency of endocytosis and increased the intracellular DOX content, while PSMA-617 in nanomedicine has little effect on the IC_50_ for PC3 cells with low expression of PSMA (Supplementary Figures S10, S11, and S13). It further illustrated that the high expression of PSMA on the cell surface was necessary for maintaining the advantage of P-SNPs@DOX.

## Conclusion

In conclusion, a targeted drug delivery system was prepared successfully to enhance therapeutic effects on prostate cancer by using host–guest molecular recognition that could self-assemble into NPs in aqueous solution. According to the supramolecular strategy, PSMA-617 was inserted into CB [8] by non-covalent self-assembly, avoiding complex synthesis. Based on the targeting capability to prostate cancer cells through receptor-mediated endocytosis, P-SNPs@DOX released more DOX inside PSMA-positive prostate cancer cells, thereby increasing the anticancer efficacy. This study provided a progressive design to introduce targeted groups into anti-cancer drugs, which has a good prospect of clinical applications and translations.

## Data Availability

The original contributions presented in the study are included in the article/Supplementary Material, further inquiries can be directed to the corresponding authors.

## Abbreviations

CB [8], cucurbit[8]uril; CLSM, confocal laser scanning microscopy; CT, charge transfer; DLS, dynamic light scattering; DOX, doxorubicin; EPR, enhanced permeability and retention; IC_50_, half maximal inhibitory concentration; ITC, isothermal titration calorimetry; K_α_, association constants; MTT, 3-(4′,5′-dimethylthiazol-2′-yl)-2,5-diphenyl tetrazolium bromide; MV, methylviologen; MWCO, molecular weight cutoff; Nap, 2-amino-3-(naphthalen-1f-yl)propanoic acid; PBS, phosphate buffer saline; PCL, poly(ε-caprolactone); PEG, polyethylene glycol; PSMA, prostate-specific membrane antigen; PTX, paclitaxel; TEM, transmission electron microscopy.
