# Supplementary material for: PSMA-Targeted Supramolecular Nanoparticles Prepared From Cucurbit[8]uril-Based Ternary Host–Guest Recognition for Prostate Cancer Therapy
Source: Front Chem. 2022 Feb 10;10:847523. doi: 10.3389/fchem.2022.847523 (PMC8867089; doi:10.3389/fchem.2022.847523)
Supplement: Supplementary file 1 [file DataSheet1.docx]

***Supplementary Material***

**Figure S1** ^1^H NMR spectrum (D_2_O, room temperature) of MV.

**Figure S2** ^1^H NMR spectrum (D_2_O, room temperature) of Nap.

**Figure S3** ^1^H NMR spectrum (D_2_O, room temperature) of CB[8].


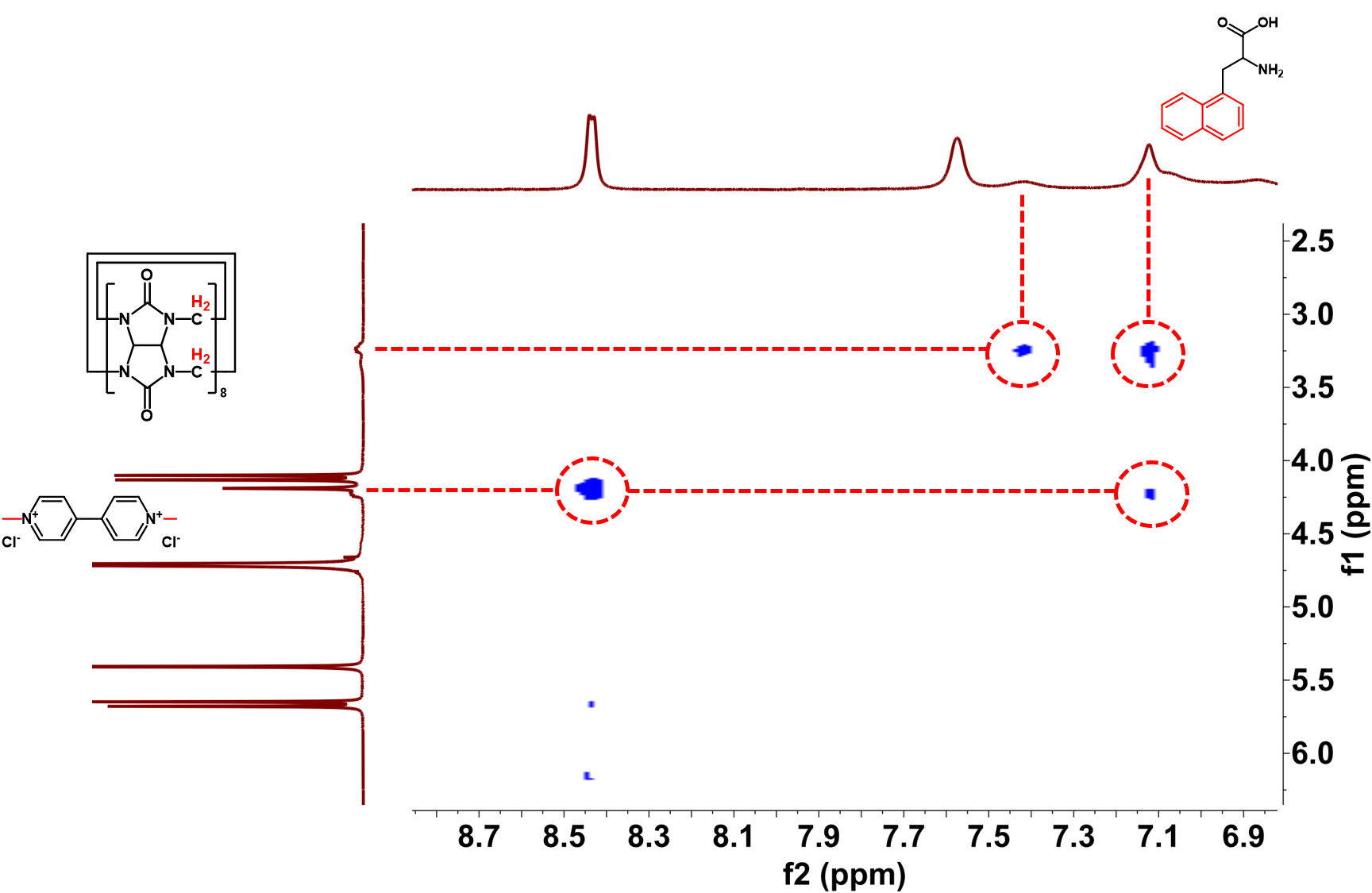


**Figure S4** 2D NOESY NMR of MV, Nap and CB[8].

**Figure S5** Cytotoxicity of SNPs against 22RV1 cells after 48 h incubation.

**Figure S6** Cytotoxicity of SNPs against PC3 cells after 48 h incubation.


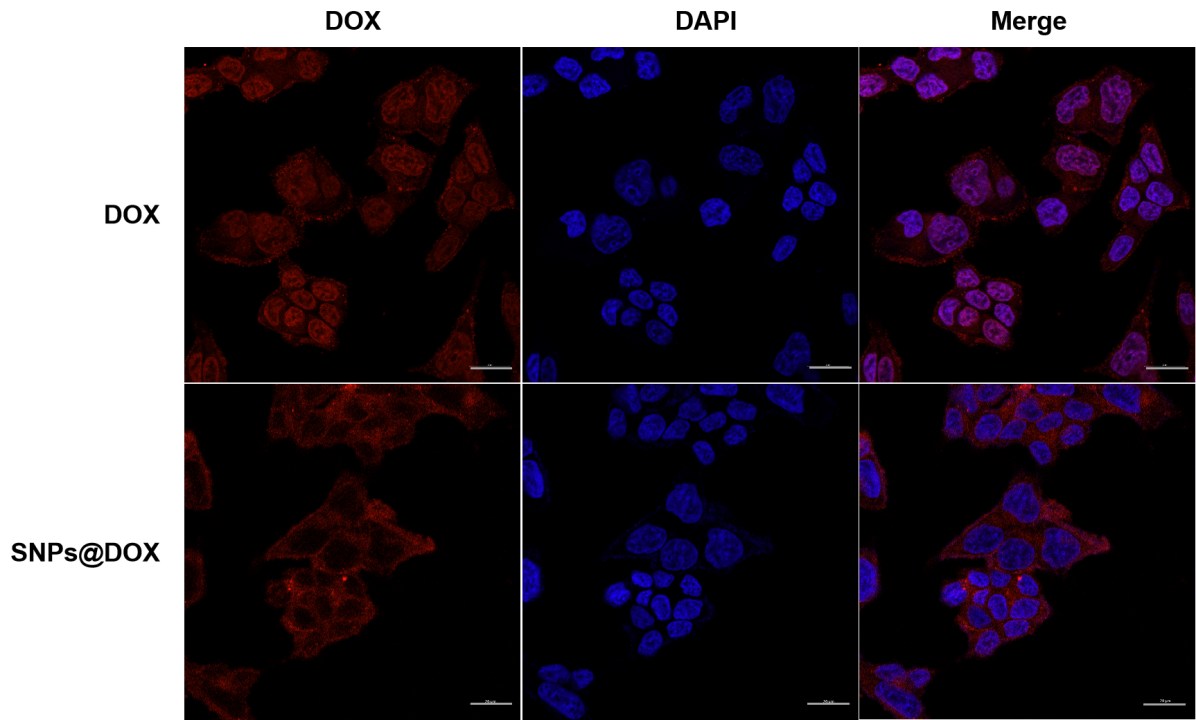


**Figure S7** CLSM images of 22RV1 cells cultured with DOX and SNPs@DOX after 4 h incubation, respectively.


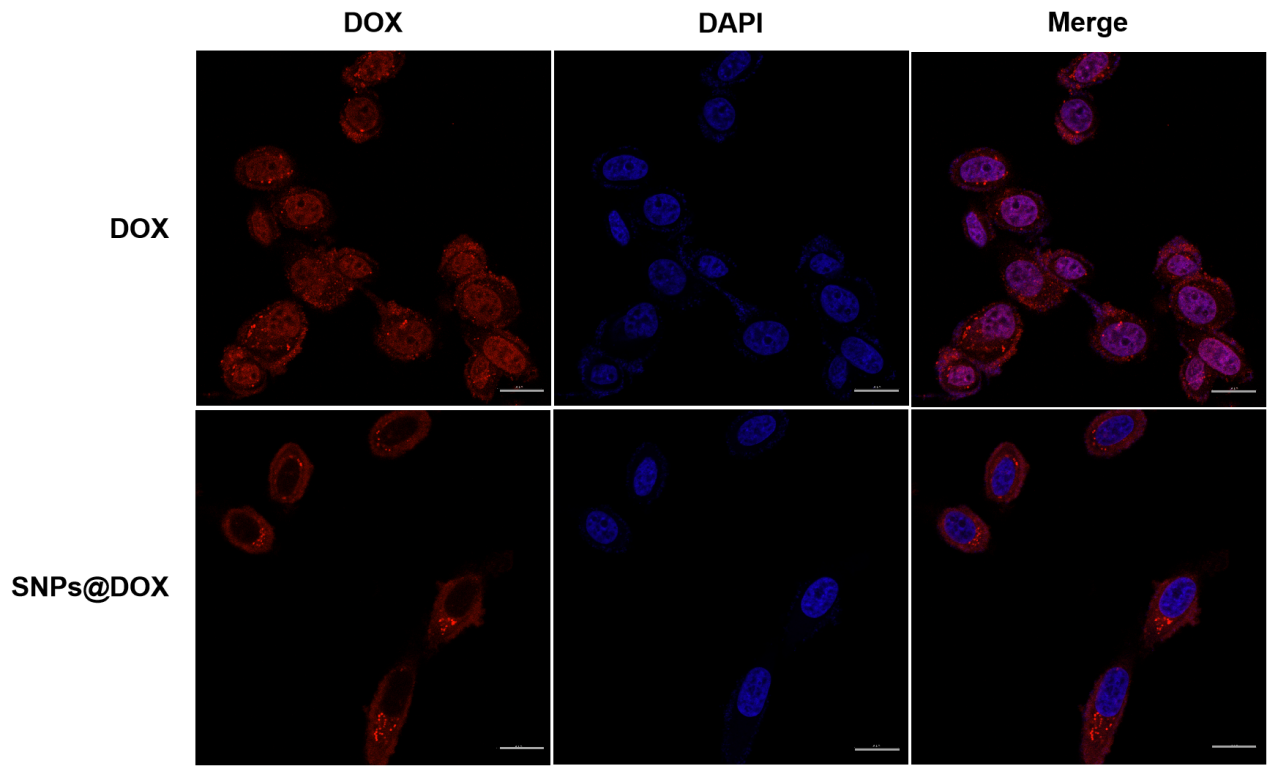


**Figure S8** CLSM images of PC3 cells cultured with DOX and SNPs@DOX after 4 h incubation, respectively.

**Figure S9** Cytotoxicity of DOX, SNPs@DOX and P-SNPs@DOX against 22RV1 cells after 24 h incubation, respectively.

**Figure S10** Cytotoxicity of DOX, SNPs@DOX and P-SNPs@DOX against PC3 cells after 24 h incubation, respectively.

**Figure S11** Cytotoxicity of DOX, SNPs@DOX and P-SNPs@DOX against PC3 cells after 48 h incubation, respectively.

**Figure S12** IC_50_ of SNPs@DOX and P-SNPs@DOX against 22RV1 after 24 h and 48 h incubation, respectively.

**Figure S13** IC_50_ of SNPs@DOX and P-SNPs@DOX against PC3 after 24 h and 48 h incubation, respectively.


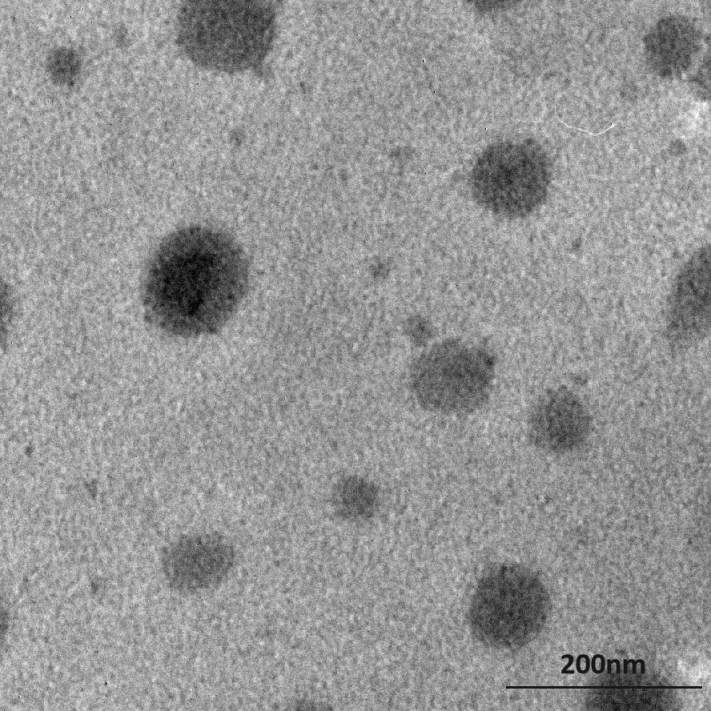


**Figure S14** TEM image of SNPs@PTX.

**Figure S1****5** Cytotoxicity of PTX and SNPs@PTX against 22RV1 cells after 48 h incubation.

**Figure S16** Cytotoxicity of PTX and SNPs@PTX against PC3 cells after 48 h incubation.
